# 

**Published:** 1890-09-13

**Authors:** 


### NOTICE TO CORRESPONDENTS.

All MS., Letters, Books for Review, and other matters intended for the Editor should be addressed The Editor, The Lodge, Porchester Square, London, W.

The Editor cannot undertake to return rejected M.S., even when accompanied by stamped directed envelope.

### Notice to Advertisers and Subscribers.

All Advertisements, Orders for Copies of The Hospital, and Business Communications should be addressed to The Publisher, *not to the Editor*, The Hospital, 140, Strand, W.C.

### Bound Volumes of "The Hospital."

Vol. VI., containing full page portraits of T.R.H. the Prince and Princess of Wales, and Miss Florence Nightingale, is obtainable at 4s., post free.

Orders will be received for Vol. VII., 4s. post free. Cases for binding may be had of the Publisher, at 1s. 6d. each.

### To Residents, Secretaries, and Matrons.

The Editor will be glad to have the Regulations and each Report as issued by Asylums, Hospitals, Convalescent and Nursing Institutions, as well as those of other Charities, and an early copy in duplicate of all Appeals and Festival Notices, etc., addressed to *The Editor, The Lodge, Porchester Square, London, W.* Any superintendent, secretary, matron, or other chief official who regularly sends such information may be registered, on application to the Publisher, to receive free of cost a cover for binding The Hospital in half-yearly volumes.

### BURDETT'S HOSPITAL ANNUAL FOR 1890

Is now ready, and may be obtained of the Publisher, The Hospital Offices, 140, Strand, W.C., price 2s. 6d. limp boards, or 3s. 6d. cloth. All orders must be accompanied by a remittance.

### General Charities.

[This Column is devoted to an account of work of charitable institutions other than Medical Charities. The Editor will be glad to receive reports or news of work at 140, Strand. *Philanthropy* should be plainly written on the envelope.]

While, on the one hand, there are many little sick children in London whose parents are perfectly indifferent as to whether they live or die, there are others who suffer simply because their parents have neither the knowledge nor the means to relieve their sufferings. Take, for instance, a very poor child with spine disease, who has nothing to do, who is unable to walk. What a cheerless, miserable little life! So it is a comfort to anybody meeting with such a case to know that the **INVALID CHILDREN'S AID ASSOCIATION**, 18, Buckingham-street, Strand, will do anything in its power to assist the child. The matron of a hospital writes, perhaps, to the secretary, and says a child is leaving, but that without constant assistance it will never regain complete health. A lady visitor then goes to the child, and looks after it in every way. Perhaps it needs nourishing food, or an invalid carriage, or a short stay at the seaside. Whatever it wants to help it is forthcoming. The children are so grateful for being made comfortable, and look forward to their visitor coming to see them and cheer them up. Hundreds of people have never heard of this work, and would gladly assist either by funds or personal help. A visit to the office is, perhaps, the most satisfactory way of finding out all about it, and the spinal carriages, crutches, and different appliances to be seen there tell their own tale of how much suffering there is among the children, which is daily needing relief. Some of the first medical men have publicly acknowledged the good done by this society, which is sufficient testimony of its efficiency.

The new St. Pancras Workhouse is to be built very plainly, so that there shall be better arranged classification. There will be more room for married couples and larger accomodation for that unhappy being the chronic loafing pauper.

The promoters of the concert, which Lady McKenna was kind enough to have at her house on the 14th ult., for the **HOME OF REST FOR HORSES** have sent £90 to that Institution.

The Surrey Hills make a splendid point of observation for watching the amount of good done to the small London children who come down for a holiday. There is something so especially free and invigorating about these breezy commons. Covered with purple heather, with a view right away to the South Downs, no lovelier spot could be found for the weary little City child. The first day when they arrive they don't eat very much, even a plentiful supply of whortlebury jam does not prove sufficiently alluring, but wait till three or four days have been passed on the hills; see the children coming home then, like a small pack of wolves, too, ready to devour anything and everything. There is so much that is new to them. "Do you dig the potatoes up out of the dirt?" asked a small fry; "we never do that in London." We saw a small child recently with a little bit of blackberry bush, wrapped up in a handkerchief, and some earth. She remarked that she was going to see how blackberries would grow in London, as her mother had a yard at the back. Seeing is believing: day by day as they pass our gate we watch the children looking better, and Oh! they are so sorry to leave the country, but "'praps we shall come next year again" is a great source of comfort to them. While some are pleading for three weeks to be the time for "a country holiday," we, while of course readily acknowledging that increased sojourn must mean increased strength, ask charitable folk to remember that "the good of the greatest number" ought to be the first consideration. It is hard on the children, and there are thousands of them, who have never breathed a mouthful of pure air, to be left at home because others luckier than themselves are getting three weeks. It is hard on them in more ways than one; they lose so much new joy, and they lose the purer thoughts which nature would give them in spite of themselves. No! Let us first bring all the children, every one of them, into the lovely fresh country, and then, when we have succeeded in that, let us begin to think about extending the holiday.

Success has crowned the efforts of those who started the **SEASIDE CAMP FOR WORKING BOYS** last year. Colonel Farquharson, late Royal Marines, commands it, and the number received this year has already exceeded last year's by a hundred. Each boy pays 2s. 6d. a week for himself, and they come recommended by their employers, subscribers, or clergy. Funds are getting low, and the Committee are most anxious that the work hitherto so successful shall not be crippled. If people would remember that the smallest help is welcome, and that if they can only send a shilling it is better than nothing at all, we should seldom hear their constant appeals for pecuniary assistance. All contributions to be sent to Seaside Camp [illegible] Working Boys, Messrs. Cocks, Biddulph, and Co., 43, Charing Cross.

St. Matthew's, Stepney, is shortly going to open a "Dockers' Palace," which sounds a trifle magnificent, but it is really to be a polytechnic for the use of the dockers in that neighbourhood.

The late Lord Magheramorne bequeathed £200 to the Royal School for Officers' Daughters, and the Soldiers' Daughters' Home at Hampstead, respectively.

